# Ewing’s sarcoma as second malignancy following a short latency in unilateral retinoblastoma

**DOI:** 10.1007/s10195-011-0152-0

**Published:** 2011-08-09

**Authors:** Naveen Tahasildar, Vijay Goni, Kishan Bhagwat, Sujit Kumar Tripathy, Bijnya Birajita Panda

**Affiliations:** 1 Department of Orthopaedics, Postgraduate Institute of Medical Education and Research, Sector-12, Chandigarh, 160012 India; 2 Department of Ophthalmology, Government Medical College, Bhubhaneshwar, India

**Keywords:** Ewing’s sarcoma, Second malignancy, Unilateral retinoblastoma, Short latency

## Abstract

Second malignancies, mostly in the form of bone sarcomas, are known to occur in hereditary retinoblastomas, which usually present with bilateral disease. Only 2 cases of Ewing’s sarcoma have been reported in the literature following sporadic unilateral retinoblastoma. A 5-year-old boy presented to our hospital with Ewing’s sarcoma of the right humerus (proven by biopsy and immunohistochemistry) following successful treatment of retinoblastoma of the left eye with enucleation and chemotherapy 2 years previously. He was treated with 2 cycles of chemotherapy followed by radiation therapy. At 15 months follow-up, the tumor had reduced in size and the child had a good functional outcome. The cumulative risk of second malignancies in retinoblastoma survivors is 32%. Ninety-eight percent of second malignancies occur in patients with bilateral retinoblastoma. Germ line mutations have been considered in sporadic tumors occurring bilaterally and multifocal unilateral sporadic tumors. Bone and soft tissue sarcomas are the most common second malignancies. Radiation therapy increases the risk of developing a second malignancy in the irradiated field. Unilateral retinoblastomas, which comprise the majority of retinoblastomas, are not immune from the development of second malignancies. Close follow-up of all retinoblastomas—even in the early period—can improve the outcome by facilitating the early detection and aggressive treatment of second malignancies.

## Introduction

Retinoblastoma (RB) is the most common malignant ocular tumor in the pediatric age group, occurring in 1/15,000 to 1/30,000 live births [16], although it forms a smaller percentage of all pediatric malignancies (~5/1,00,000 children) [9]. There are two broad subgroups—hereditary (40%) and nonhereditary (60%) [10]. The hereditary group present at a younger age, usually have bilateral disease, and have an underlying germ line mutation of the RB1 gene. The nonhereditary group present at a later age, and have unilateral disease as well as an underlying genetic mutation arising in the somatic cells. More than 90% of these patients experience long-term survival due to successful treatment regimens [1]. Both radiation therapy and chemotherapy with alkylating agents increase the risk of subsequent development of bone sarcomas in children who survive childhood cancers [17]. Nonocular second malignant neoplasms have occurred almost exclusively in children with bilateral disease, who constitute only 25% of cases. The most common second malignant neoplasms in these patients are sarcomas, especially bone sarcomas, though a wide variety of second tumors have been reported, such as melanoma, chondrosarcoma, leukemia, neuroblastoma, and leiomyosarcoma [7]. Bone and soft tissue sarcomas are the most common second malignant neoplasms; osteogenic sarcoma is the single most common second malignant neoplasm [7].

Previously, six cases of Ewing’s sarcoma in patients with a prior history of bilateral [1, 6, 8, 16] and two cases of unilateral [6, 12] retinoblastoma have been reported. Here, we present a case of a child with unilateral RB who developed Ewing’s sarcoma of the right humerus 2 years after enucleation of the left eye, and a review of the pertinent literature.

## Case report

**Fig. 1:**
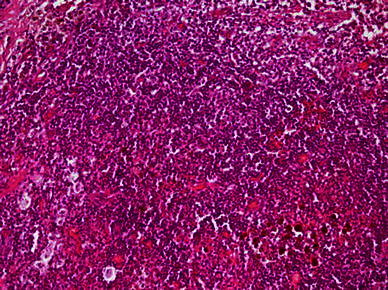
Low-power view showing malignant small blue round blue tumor cells, a few macrophages with hemosiderin pigmentation, and occasional rosette formation (stain: hematoxylin and eosin; original magnification, × 20)

A 3-year-old male child was brought to the eye center of our hospital with a yellow reflex in the left eye (cat’s eye reflex), and was found to have retinoblastoma of an advanced stage. He was subsequently fully investigated and found to have nonmetastatic locally advanced retinoblastoma. Since there was no vision in the left eye, enucleation was performed as definitive treatment and a specimen was sent for histopathological analysis. It showed degeneration of all layers of the eyeball, with areas of calcification, bone formation and chronic inflammatory infiltrates. Focally, a tumor with a collection of small round blue cells with a high nucleo/cytoplasmic ratio and hyperchromatic nuclei consistent with retinoblastoma was evident (Fig. 1). The optic nerve resection limit was free of tumor. The patient was started on a carboplatin, vincristine and etoposide chemotherapy protocol postoperatively.

**Fig. 2:**
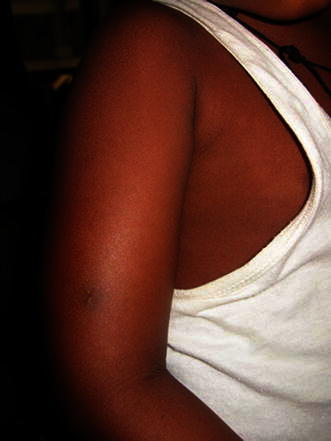
Clinical photograph of the right arm of the child showing diffuse swelling extending from the proximal third to just above the elbow

At the age of 5 years, the child was brought to the orthopedic clinic with complaints of painful swelling of the right arm that had been present for 1 month, which was gradually increasing in size (Fig. 2). The pain was reported to increase at night and was relieved partially by analgesics. Local examination revealed a 10 × 4 cm fusiform swelling involving the distal shaft of the humerus circumferentially. A local rise in temperature and tenderness was present over the swelling. Terminal flexion of the elbow was restricted by 30°.

**Fig. 3:**
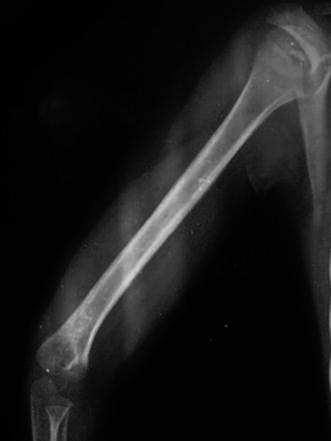
Roentgenogram of the right humerus showing a diffuse periosteal reaction with a permeative type of destruction involving almost the whole of the diaphysis and a huge soft-tissue shadow surrounding the diaphysis

**Fig. 4:**
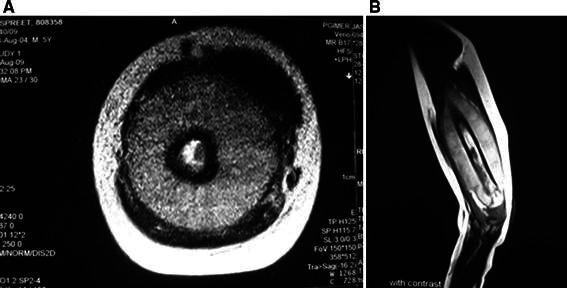
**a** Axial section at the mid-shaft humerus after gadolinium-enhanced MRI, showing circumferential, moderately enhancing, well-marginated peridiaphyseal soft-tissue mass. **b** Coronal section of the humerus after gadolinium-enhanced MRI showing an intramedullary altered signal with moderate enhancement involving almost the whole of the diaphysis

Roentgenograms revealed a lamellated pattern of periosteal reaction and a permeative pattern of osteolysis involving the distal shaft of the humerus with soft-tissue shadows (Fig. 3). Gadolinium-enhanced MRI of the part showed a circumferential, moderately enhancing, well-marginated peridiaphyseal soft-tissue mass involving the humeral diaphysis from the proximal third to the distal metaphysis (Fig. 4a, b).

A whole-body bone scan showed no evidence of bony metastasis. However, contrast-enhanced computed tomography (CECT) of the chest showed two pulmonary nodules in the right lung, suggestive of metastasis. A guided biopsy of the nodules confirmed the diagnosis of metastasis.

**Fig. 5:**
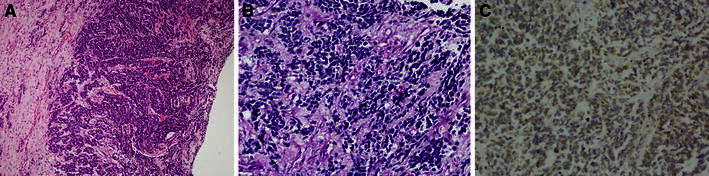
**a** Low-power view showing tumor cells infiltrating into soft tissue in a cord-like pattern (stain, hematoxylin and eosin; original magnification, × 20). **b** High-power view showing malignant blue round tumor cells with scanty cytoplasm and dispersed chromatin along with many mitotic figures showing PAS positivity and conspicuously absent rosette formation, indicating a poorly differentiated high-grade tumor (stain, hematoxylin and eosin; original magnification, × 40). **c** Immunohistochemistry showing tumor cells with Mic-2 positivity but which are negative for neuron-specific enolase (stain, immunohistochemical; original magnification, × 20)

Open biopsy of the tumor was planned as part of the staging studies. Biopsy revealed small round tumor cells arranged in sheets, infiltrating into the skeletal muscle. Cells were small with hyperchromatic nuclei, inconspicuous nucleoli and a high nuclear-to-cytoplasmic ratio. No rosette formation or Azzopardi phenomenon was seen. This was an indication of a high-grade tumor with a poor immunologic/host response. Immunohistochemical staining for cell-surface glycoprotein p30/32^MIC2^(CD99) was positive, and neuron-specific enolase was negative, strongly suggesting a diagnosis of Ewing’s sarcoma, excluding the possibility of metastasis following retinoblastoma, and ruling out other small round cell tumors (Fig. 5a, b, c).

The patient was classified as being at an advanced stage of the disease due to the presence of metastasis. Treatment was directed towards palliating symptoms at the local site by radiotherapy and aggressive chemotherapy. Since the patient was already being treated with a standard chemotherapy protocol, a modified chemotherapy regimen with ifosfamide and etoposide was undertaken. The patient underwent 2 cycles of chemotherapy with ifosfamide and etoposide followed by radiotherapy in 2 phases, initially with 44 Gy and subsequently with 20 Gy. The patient was followed up regularly for 18 months. By that time, the swelling decreased in size and was not tender. Complete elbow range of motion was regained, and the vision in the right eye was normal.

## Discussion

Survivors of retinoblastoma invariably carry a high risk of developing a second malignant neoplasm, with the cumulative risk being 32% [1]. Ninety-eight percent of the secondary malignant tumors occur in patients with bilateral retinoblastoma or in 15% of patients with unilateral RB with underlying germline mutation. However, unilateral RBs comprise almost 75% of all retinoblastomas [1]. By far the most common second malignant neoplasm has been osteosarcoma [15]. The second most frequent second malignant neoplasms have been soft-tissue sarcomas [3].

In children previously irradiated for retinoblastoma, 70% of second malignant neoplasms have occurred in the field and 30% outside the field of radiation [1]. Abramson et al. [1] reviewed 2,302 survivors of childhood retinoblastomas. 71.3% of neoplasms occurred within the radiation therapy field after an average latent period of 11.4 years, and 18.8% occurred outside the radiation field after an average latent period of 11.1 years. Osteosarcoma was the most common second malignant neoplasm, irrespective of the relation between radiation therapy field and location of the tumor. Roarty et al. [14] evaluated 215 patients for the cumulative incidence of second neoplasms in patients with bilateral retinoblastoma using life table methods. Second tumors developed in 4.4% of the patients during the first 10 years of follow-up, 18.3% after 20 years, and 26.1% after 30 years. In their group of patients, the 30-year cumulative incidence was 35.1% for the 137 patients who received radiation therapy, compared with 5.8% of 78 patients who did not receive radiation therapy. There was a 30-year incidence rate of 29.3% for second tumors within the field of irradiation, and 8.1% outside the field. These findings suggested that carriers of the retinoblastoma gene have an increased incidence of second tumors and that the incidence rate is further increased in patients who received radiation therapy.

Heritable retinoblastoma and osteosarcoma were found to be associated in some cases with the deletion of the 13q14 locus of the RB-1 gene [5]. A variety of second malignant neoplasms have been reported in the literature, including osteosarcoma, fibrosarcoma, skin carcinomas, malignant melanomas, rhabdomyosarcomas, acute lymphoblastic leukemia, and sinonasal carcinoma, but few reports indicate the frequency of development of Ewing’s sarcoma after retinoblastoma [6]. Clinically, it is difficult to differentiate a unilateral retinoblastoma of heritable type from nonheritable but generally sporadic tumors occurring bilaterally, and multifocal unilateral sporadic tumors are considered germ cell mutations [16]. Patients with unilateral, unifocal retinoblastoma and negative family histories have not been considered to be at increased risk for second malignant neoplasms. Although, to the best of our knowledge, the current report is the third (after Helton et al. [6] and Mittal et al. [12]) on Ewing’s sarcoma in unilaterally affected patients with retinoblastoma, it is not possible to exclude the presence of a germinal mutation in any of these cases.

Skeletal scintigrams are not economically feasible to use for screening purposes to detect second malignant neoplasms, despite their capacity for earlier detection [13]. MRI is a very sensitive method of displaying bone lesions but, in the absence of localizing symptoms, it is not yet a practical method of routine skeletal imaging.

Children of affected parents have a 50% risk of having retinoblastoma [4]. The risk of second malignant neoplasms in retinoblastoma has been variably estimated to be between 1.5 and 90% [11, 16]. This increased incidence is believed to be secondary to the loss of normal tumor suppression activity of the retinoblastoma gene on chromosome 13. Radiation or chemotherapy or both put these patients at further risk, though second malignant neoplasms are also common in patients who have not received these adjuvant treatments. Patients with the genetic form of retinoblastoma are also at higher risk.

Our patient developed a second malignancy 2 years after the initial diagnosis of RB, but this period is much shorter than those described in previous reports (Table 1): 4.3 years [4], 14.2 years [4], and 18 years (range 10–32 years) [2]. The clinical outcome of children who develop a second malignancy depends upon the location and response of the second tumor to treatment, and aggressive multi-modality therapy is the key [2]. Our patient who underwent very aggressive treatment, which included induction chemotherapy, surgery and high-dose chemotherapy, is surviving and being followed up very closely.

**Table 1:** Ewing’s sarcoma as a second malignant neoplasm after retinoblastoma:literature review

Sl. no.	Author [reference]	Year of publication	Unilateral/bilateral retinoblastoma	Latent period	Age of presentation	Other remarks
1	Kitchen [8]	1976	Bilateral	–	–	–
2	Kitchen [8]	1976	Bilateral	–	–	–
3	Schifter et al. [16]	1983	Bilateral	9 yrs	–	–
4	Abrahamson et al. [1]	1984	Bilateral	–	–	RT given
5	Abrahamson et al. [1]	1984	Bilateral	–	–	RT given
6	Helton et al. [6]	1993	Bilateral	12.5 yrs	13.1 yrs	Chemoradiotherapy given
7	Helton et al. [6]	1993	Unilateral	18.25 yrs	21 yrs	Death due to metastasis
8	Mittal et al. [12]	2008	Unilateral	3 yrs	4 yrs	Chemotheray + surgery + autologous stem cell transplantation

The present case highlights the fact that patients with retinoblastoma are at an increased risk of developing a second malignant neoplasm, the latency of which is highly variable. It is also worth noting that unilateral retinoblastoma, which represents the majority of cases, is not immune from the development of second malignant neoplasms, considering that a germline mutation can never be ruled out. Ewing’s sarcoma, a tumor which responds excellently to chemoradiotherapy, should not be missed in the primary diagnosis of bone sarcomas presenting as second malignant neoplasms. Close follow-up, early detection and aggressive treatment of second malignant neoplasms can improve the outcome.

## Ethical considerations

Written consent was obtained from the parents of the child before submission of this case report and of any accompanying images.
